# Maladie de kaposi classique à localisation palpébrale

**DOI:** 10.11604/pamj.2016.24.84.8712

**Published:** 2016-05-26

**Authors:** Radia Chakiri, Fatima Zohra Mernissi

**Affiliations:** 1 Service de Dermatologie, CHU Hassan II de Fès, Maroc

**Keywords:** Maladie de kaposi, classique, paupière, Kaposi's disease, classical, eyelid

## Image en médecine

La maladie de kaposi classique est une affection proliférative et multifocale à double composante vasculaire et cellulaire fibroblastique, d'expression cutanée et viscérale. Nous rapportons le cas d'une femme de 70 ans présentait depuis 6 mois des lésions érythémateuses au niveau des 2 paupières supérieures gênant la vision. L'examen clinique trouvait 2 nodules érythémato-violacé de bord libre des 2 paupières supérieures, plaques et papules érythémato-violacées des membres supérieures et inférieures. L'examen histologique de la biopsie de nodule palpébrale objectivait: une prolifération endothéliale vasculaire et fuso-cellulaire en faveur de la maladie de kaposi. La sérologie HIV faite était négative. La patiente avait bénéficié d'une exérèse chirurgicale des lésions palpébrales avec un recul de 1 an. La maladie de Kaposi classique affecte la population du bassin méditerranéen et de l'Europe de l'Est, surtout les hommes de plus de 50 ans. Les localisations oculaires intéressent les paupières, la conjonctive, le sac lacrymal et plus rarement l'orbite. Elles surviennent au cours d'une maladie de Kaposi déjà connue ou, plus rarement, constituent le premier signe de l'affection. Leur fréquence, rare avant l'apparition du SIDA. Les localisations palpébrales touchent les paupières supérieures et inférieures dans 15% des cas. Le diagnostic différentiel de ces formes palpébrales doit être fait avec les carcinomes baso-cellulaires, les blépharites et les orgelets. Les complications des localisations palpébrales sont représentées par l'entropion, l'ectropion, le trichiasis, d'irritations oculaires, d'ulcère et d'abcès cornéen ou d'hémorragies conjonctivales. La prise en charge est chirurgicale car les lésions palpébrales sont toujours gênantes sur le plan fonctionnel.

**Figure 1:**
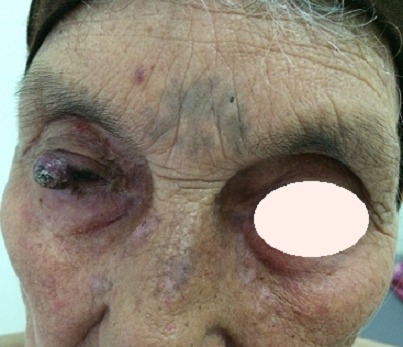
Nodule érythémateux violacé du bord libre de la paupière supérieure droite

